# Effects of the COVID-19 pandemic on prehospital emergency care for stroke and transient ischaemic attack: A protocol for a systematic review and meta-analysis

**DOI:** 10.12688/hrbopenres.13534.1

**Published:** 2022-03-25

**Authors:** Edel Burton, Vera JC McCarthy, Aine Merwick, Patricia M Kearney, Claire M Buckley

**Affiliations:** 1 School of Public Health, University College Cork, Cork City, Cork, T12XF62, Ireland; 2 School of Nursing and Midwifery, University College Cork, Cork City, Cork, T12AK54, Ireland; 3 Department of Neurology, Cork University Hospital, Cork City, Cork, T12 DC4A, Ireland; 4 Office of the NCAGL for Chronic Disease, Health Service Executive South East, Lacken, Dublin Road, Kilkenny, R95 NV08, Ireland

**Keywords:** Ambulance response times; emergency care; COVID-19 pandemic; prehospital; protocol; stroke; systematic review; transient ischaemic attack.

## Abstract

Background

The COVID-19 pandemic impacted on health service provision worldwide, including care for acute time sensitive conditions. Stroke and transient ischaemic attacks (TIA) are particularly vulnerable to pressures on healthcare delivery as they require immediate diagnosis and treatment. The global impact of the COVID-19 pandemic on prehospital emergency care for stroke/TIA is still largely unknown. Thus, the aim of this study is to conduct a systematic review and meta-analysis to investigate the impact of the COVID-19 pandemic on prehospital emergency care for stroke and TIA.

Methods

Following the Preferred Reporting Items for Systematic Reviews and Meta Analyses (PRISMA) guidelines, the review is registered on PROSPERO (registration number CRD42022315260). Peer-reviewed quantitative studies comparing prehospital emergency care for adults with stroke/TIA before and during the COVID-19 pandemic will be considered for inclusion. The outcomes of interest are ambulance response times and emergency call volumes for stroke/TIA. A systematic search of databases including PubMed, Embase and Scopus will be conducted. Two authors will independently screen studies for inclusion based on predetermined inclusion and exclusion criteria. Data extraction and quality assessment will be conducted by two authors. Meta-analysis will be performed to calculate overall pooled estimates of ambulance response times (primary outcome) and stroke/TIA call volumes (secondary outcome), where appropriate.  Where heterogeneity is low a fixed-effects model will be used and where heterogeneity is high a random-effects model will be used. Subgroup and sensitivity analyses will include location, stroke/TIA diagnosis and COVID-19 case numbers.

Results

Data on primary and secondary outcomes will be provided. Results of subgroup/sensitivity analyses and quality assessment will also be presented.

Conclusions

This review will identify existing literature reporting the impact of the COVID-19 pandemic on prehospital emergency care for adult patients with stroke/TIA and provide summary estimates of effects on ambulance response times.

## Introduction

The COVID-19 pandemic was a “shock” to the health system, globally ^1^. Consequently, it is reported that the pandemic affected non-COVID healthcare in many countries ^2, 3^. Public health guidelines were introduced in an effort to manage the pandemic, including travel restrictions and stay at home orders. These interventions may have impacted on healthcare seeking behaviours. Furthermore, the healthcare workforce was directly impacted through sickness and periods of isolation/restriction of movements for cases and contacts ^4^.

Globally, delayed, and reduced admissions for non-COVID related care have been linked to increased mortality and morbidity ^5, 6^. Reports from multiple countries indicated that calls to emergency medical services vastly increased over the course of the pandemic ^7^. As a result, further pressure was put on prehospital emergency services ^7^.

The prehospital phase of healthcare is defined in a World Health Organisation report as the period before arrival at a hospital, clinic, and other fixed healthcare setting ^8^. Prehospital care generally includes the provision of care by emergency medical service providers such as emergency medical dispatchers, emergency medical responders, emergency medical technicians, and paramedics ^9^. As ambulance response times are internationally recognised key performance indicators for prehospital emergency care they will be used as the primary outcome of interest in this review ^10^.

In 2019, stroke was the second leading cause of disability-adjusted life-years (DALYs) globally, in the 50–74 years and 75+ years age groups ^11^. Up to one in three strokes are preceded by a transient ischaemic attack (TIA), with approximately 50% of these occurring within a year after the TIA ^12^. Stroke is a medical emergency and requires immediate evaluation, confirmation of diagnosis and treatment in order to prevent brain damage ^13^. Early diagnosis and treatment are also imperative for TIA, to reduce mortality and risk of stroke ^14^. Due to the requirement for immediate care with stroke and TIA, they are particularly vulnerable to pressures on health system care delivery or changes in care seeking behaviours by patients. Recent preliminary evidence suggests that stroke and cardiac arrest were the emergency cases most affected by the COVID-19 pandemic ^7^.

There has been a global decrease in the number of patients seeking medical care for stroke and TIA during the pandemic ^15– 17^. Thus, COVID-19 has potentially had a disruptive effect on the stroke chain of survival ^6, 18^. It has been reported that stroke admissions in Southern Europe have fallen by 25% over the pandemic period ^17^. Furthermore, the number of emergency medical service calls dispatched to stroke dropped, and a 30-minute delay in response times have been reported, in this region ^17^. One narrative review stated that the suggested disruption in the emergency stroke care pathway due to the COVID-19 pandemic has resulted in a global surge of prehospital mortality ^7^.

There has been some research done in this area ^7, 19– 21^. However, it is still largely unknown what impacts on prehospital emergency care for stroke and TIA were seen and how they varied in different countries, with different approaches to the management of the pandemic and different underlying healthcare systems. Thus, this systematic review and meta-analysis aims to summarize the existing international literature on the impact of the COVID-19 pandemic on prehospital emergency care for adult patients with stroke or TIA and estimate the ambulance response times and emergency call volumes for stroke/TIA.

## Protocol

### Methods and design

This protocol was developed using the Preferred Reporting Items for Systematic Reviews and Meta-Analysis Protocols Checklist (PRISMA-P) ^22^. The proposed systematic review and meta-analysis will follow the Preferred Reporting Items for Systematic Reviews and Meta Analyses (PRISMA) guidelines ^23^. This review is registered on PROSPERO ( registration number CRD42022315260).

### Aim

To summarize the existing international literature on the impact of the COVID-19 pandemic on prehospital emergency care for adult patients with stroke or TIA and estimate the ambulance response times and emergency call volumes for stroke/TIA.

### Objectives

● To investigate if ambulance response times (activation times, response times and patient care times) for patients with stroke/TIA differed before and during the COVID-19 pandemic.
● To investigate if the volume of emergency services calls for stroke/TIA differed before and during the COVID-19 pandemic.

### Participants

Adult patients (≥18 years of age) with stroke or TIA.

### Exposure

Prehospital emergency care for stroke/TIA during the COVID-19 pandemic.

### Comparison

Prehospital emergency care for stroke/TIA prior to the COVID-19 pandemic.

### Outcome

Primary outcomes: activation time, response time, patient care time.

Secondary outcome: emergency medical services call volume for stroke/TIA.

### Ambulance response times

Ambulance response times include three main time periods in the time from receipt of the emergency call by the call centre operator to the patient arriving at the hospital ^24^. **“Activation time “**covers the period from receipt of the call to mobilisation of a fully crewed emergency ambulance ^24^. **“Response time”** covers the period from receipt of call to the arrival of the ambulance at the scene of the emergency ^24^. **“Patient care time”** refers to the time from arrival of an ambulance crew at the scene to arrival at hospital ^24^. The terminology used to describe these three key time periods can vary between countries and publications ^7, 24^. Regardless of the term used, these three distinct periods of time are a key focus of this review. Thus, the search terms of the review relating to emergency care have remained broad to encompass as many variations as possible.

### Criteria for considering studies for the review

***Inclusion criteria***

Table 1 details full inclusion and exclusion criteria and justifications for each. This systematic review will include:

**Table 1.** Inclusion and exclusion criteria for study selection.

Criterion	Inclusion	Exclusion	Justification
Language	All languages	N/A	Translation of articles will ensure the largest pool of potentially eligible articles
Focus	Quantitative studies on the impact of the COVID-19 pandemic on prehospital emergency care for adult patients with stroke/TIA	Qualitative articles Opinion pieces	Pooled estimates can be calculated
Types of articles	Peer reviewed journal articles	Editorials or other opinion pieces Reviews Unpublished (grey) literature including theses, dissertations, editorials, and book chapters Newspapers and websites Conference abstracts/proceedings	Primary peer reviewed literature will provide numeric information related to primary and secondary outcomes for possible inclusion in meta-analysis
Geographic location	Any	N/A	COVID-19 is a global concern

● quantitative studies where prehospital care for adult stroke/TIA patients was compared before and during the COVID-19 pandemic.
● Stroke/TIA diagnosis does not have to be confirmed at hospital level. Due to the context of this study stroke/TIA can be suspected (based on symptoms given to call recipient) or working diagnosis after review by emergency medical services team. A study will not be excluded based on the definition of stroke/TIA diagnosis. However, if available in the study, whether the stroke/TIA was suspected or confirmed will be outlined in the review.
● Studies need to include data on ambulance response times and stroke/TIA emergency call volumes in order to be considered for inclusion. A study needs to include data on activation time, ambulance response time and patient care time to be eligible. Regardless of the terminology used in a particular study, if data were available on these three time periods of interest the study is eligible for inclusion.
● Primary, peer – reviewed studies in any language.

***Exclusion criteria***

● Studies where all participants are children, or where data for adults cannot be extrapolated.
● Case reports, case series, letters, commentaries, notes, editorials, and conference abstracts, dissertations, reviews, opinion pieces.
● Studies which report only on the secondary outcome of interest but not the primary outcome of interest.

## Search strategy for identifying relevant studies

### Bibliographic database searches

***Initial search:*** ProQuest and PubMed will be used to search for relevant articles. The librarian recommended these databases due to the context of the study, and the range of articles available on these databases. Words and phrases found in the title, abstract, and index of these papers will inform the final search strategy.

***Second search:*** Using the identified search terms a formal search of Cochrane Library (Wiley) Embase (Elsevier), ProQuest, PubMed, Scopus (Elsevier), Web of Science (Clarivate) and Wiley will be conducted. These searchers will be included in the final PRISMA flow chart ^23^.

**Reference list search:** Backwards searching of reference lists of all included studies will be carried out. The Peer Review of Electronic Search Strategies (PRESS) will be used to evaluate the search strategy ^25, 26^. The number of studies identified in the reference list screening will be included in the PRISMA flow chart ^23^.

An expert university librarian was involved in the selection of initial search terms and databases for this protocol. The librarian will advise on refining and designing the final search strategy. They will advise on the most appropriate Medical Subject Headings (MeSH) terms for the search strategy and offer input into adapting these terms for the selected databases. Table 2 details a sample search strategy for the PubMed database.

**Table 2.** Sample search terms for the PubMed database.

Initial Keywords
stroke OR cerebrovascular accident OR transient ischaemic attack OR TIA
emergency medical service OR EMS OR ambulance OR emergency care OR paramedic OR 999 OR 911 OR 112 OR prehospital
COVID-19 OR SARS-CoV-2 OR covid 19 OR coronavirus

### Selection of studies for inclusion in the review

Identified citations will be collated and uploaded into Endnote™ 2 *0 (Clarivate Analytics, PA, USA))* and duplicates removed. Titles and abstracts of published literature will be imported into Covidence ( https://www.covidence.org/)., and screened using the software, by two independent reviewers (EB and VMc) for assessment against the inclusion/exclusion criteria for the review. Potentially relevant sources will be retrieved in full, and their citation details imported into Covidence. The full text of selected citations will be assessed in detail against the inclusion criteria by EB and VMc.

Reasons for exclusion of sources of evidence at full text that do not meet the inclusion criteria will be recorded and reported in the systematic review. Any disagreements that arise between the reviewers at each stage of the selection process will be resolved through discussion between EB and VMc. If necessary, any disagreement will then be referred to a third reviewer (CB or PK) and resolved by consensus.

The results of the search and the study inclusion process will be reported in full in the final systematic review and presented in PRISMA flow diagram ^23^.

**Data extraction and management**

 A standardised extraction form has been composed using Microsoft Word (version 2102), which fulfils the eligibility criteria ( Table 1). This template has been compiled based on the aim and objective of the review and what data will be required to effectively report the results of this review.

Consistency of data extraction will be achieved, as EB will extract the data from a sample of three papers, and this will be checked by VMc. Finalising the data extraction form may be an iterative process, and modification or revision may occur after piloting. Any disagreements will be resolved upon discussion with CB and PK. This process ensures transparency and clarity in the process of data extraction. The categories below will be included in the first version of the form, which can be found in Table 3. Any modifications to the existing data extraction form will be reported in the systematic review.

**Table 3.** Categories included in the data extraction form for this systematic review and meta-analysis.

Author(s), month, year of publication, country
Aims/purpose
Study design
Country/region
Income level of country
Sample size in studies, sex, age
Activator response time before and during the pandemic
Ambulance response time before and during the pandemic
Patient care time before and during pandemic
Call volumes before and during the pandemic
Time period (pre and during COVID-19 pandemic)
Method of stroke/TIA diagnosis
COVID-19 cases numbers/ICU numbers/hospitalisations

### Appraisal of the quality of included studies

The appropriate Joanna Briggs Institute Critical Appraisal tool ^27^ will be used to appraise the quality of each included study. Two reviewers (EB and a second reviewer) will independently assess study quality. If necessary, discrepancies will be resolved by a third reviewer.

### Presenting and reporting the results

A PRISMA flow diagram ^23^ will be included in the review to illustrate the study selection process, and also will provide a rationale for excluding studies. Tables displaying study characteristics and quality assessment will be included. Forest plots will be used to present pooled estimates. If a study is eligible for inclusion in the review but does not include sufficient data for inclusion in the meta-analysis the corresponding study authors will be contacted for access to raw data, in the first instance. If raw data cannot be obtained, the findings of the relevant studies will be included in a separate table or narratively.

Meta-analysis will be conducted, where the data allows, to calculate pooled estimates of the difference between ambulance response times (time of call to ambulance being dispatched (activation time), time from ambulance being dispatched to arrival at the incident location (response time), and time from the incident location to the hospital (patient care time) and call volumes for stroke/TIA before and during the COVID-19 pandemic.

Where heterogeneity is low (I2 value of less than 50%) a fixed-effects model will be used and where heterogeneity is high (I2 value of 50% or more) a random-effects model will be used, according to the Cochrane Handbook criteria ^28^.

The following subgroup/sensitivity analyses will be performed using RevMan 5.4 where the data allow:

1. According to location.
2. According to income level of country.
3. According to study quality.
4. According to COVID-19 case numbers/hospitalisations in the country/area at the time of the study.
5. According to the number of weeks since the World Health Organisation categorized COVID-19 as a pandemic. (This was stated by the Director-General of the WHO at the media briefing on 11 March 2020 ^29^)
6. According to stroke/TIA diagnosis (suspected stroke/TIA, working diagnosis after review by emergency medical services, or hospital confirmed diagnosis)

A funnel plot will be used to assess publication bias if ten or more studies are included in the meta-analysis. Any asymmetry of the funnel plot arising from publication bias will be addressed using the trim and fill method ^30^.

If any further subgroup/sensitivity analyses need to be carried out during the meta-analysis process, these will be identified as post hoc analyses.

The quality of evidence will be assessed using the Grading of Recommendations Assessment, Development and Evaluation guidelines (GRADE) ^31^.

### Consultation with stakeholders

A Consultant Neurologist (AM) aided in the development of the research question for this review. AM will be asked about resources on the review topic that might not be identified through the searching of databases, and references. The consultant neurologist will help with dissemination of review results and offer suggestions on how best to disseminate the results of the review to the medical community.

Patient and public involvement (PPI) is described in this protocol and will be described in the systematic review using the GRIPP 2 checklist (short version) ^32^.

A PPI panel of 5 stroke survivors (2 female, 3 male) from a stroke support group were involved in the development of this protocol and subsequent review from an early stage. The PPI panel were consulted on this protocol by means of two face-to-face meetings. PPI contributors were involved in this protocol to advise on development of the research question, which stakeholders to target for involvement in the review, possible search terms, terminology surrounding stroke survivors and their research priorities.

The PPI members emphasised that they believe that the period from onset of symptoms to arrival at hospital was the most important part of the care pathway. They were asked to advise on preferred terminology around the term “stroke survivor” or “stroke patient” and any colloquial terms used for stroke or TIA. Also, they were asked what they felt would be important to know about the impact of the COVID-19 pandemic on prehospital emergency care for those with a stroke/TIA during the COVID-19 pandemic.

As a result, the research question focuses on prehospital emergency care for those with stroke/TIA. PPI had a very positive effect on this protocol ^33^. The PPI contributors used their lived experience to highlight key issues of importance and aspects of stroke care they felt could have been affected by the COVID-19 pandemic. The PPI panel prefer the term “stroke survivor” to refer to those who had a stroke. Thus, where possible this terminology will be used in outreach and dissemination of the review results, especially that targeted towards the lay population.

This group of PPI contributors will also be involved in interpreting the results of this review to identify gaps and in the dissemination of the results.

## Conclusion

This systematic review and meta-analysis will summarise existing literature investigating the impact of the COVID-19 pandemic on prehospital emergency care for those with stroke/TIA. This work may also influence policy guidelines and future research on prehospital management of non-communicable diseases during a pandemic. The findings of this review will be disseminated through peer and public presentations, conferences, a policy brief for relevant clinical programmes (stroke and emergency care) and a peer-reviewed journal.

## Study status

The protocol was registered prospectively with Prospero ( registration number CRD42022315260).

Initial searches to develop search terms and select databases had commenced, and author roles were decided at the time of publication of this protocol.

## Ethics

Ethical approval is not required for a systematic review.

## Data availability

No data are associated with this article.
