# Supplementary material for: Effects of the COVID-19 pandemic on prehospital emergency care for adults with stroke and transient ischaemic attack: A protocol for a systematic review and meta-analysis
Source: HRB Open Res. 2022 Jun 22;5:24. Originally published 2022 Mar 25. [Version 2] doi: 10.12688/hrbopenres.13534.2 (PMC9266003; doi:10.12688/hrbopenres.13534.2)
Supplement: Peer review history

# Peer review: Reviewer response for version 2

**DOI:** 10.21956/hrbopenres.14835.r32373

**Published:** 2022-07-06

**Authors:** Eithne Sexton

**Affiliations:** 1 Data Science Centre, School of Population Health, Royal College of Surgeons in Ireland, Dublin, Ireland

Many thanks to the author for their comprehensive response and revisions to the paper. I am happy that my comments have been addressed adequately and to approve the revised paper.

Is the study design appropriate for the research question?

Yes

Is the rationale for, and objectives of, the study clearly described?

Yes

Are sufficient details of the methods provided to allow replication by others?

Partly

Are the datasets clearly presented in a useable and accessible format?

Not applicable

Reviewer Expertise:

Stroke epidemiology; epidemiological modelling; health services research

I confirm that I have read this submission and believe that I have an appropriate level of expertise to confirm that it is of an acceptable scientific standard.

# Peer review: Reviewer response for version 2

**DOI:** 10.21956/hrbopenres.14835.r32375

**Published:** 2022-06-29

**Authors:** Janet E. Bray

**Affiliations:** 1 Department of Epidemiology and Preventive Medicine, Department of Epidemiology and Preventive Medicine, Melbourne, Vic, Australia

The revisions are appropriate and I am happy to approve.

Is the study design appropriate for the research question?

Yes

Is the rationale for, and objectives of, the study clearly described?

Yes

Are sufficient details of the methods provided to allow replication by others?

Yes

Are the datasets clearly presented in a useable and accessible format?

Not applicable

Reviewer Expertise:

Prehosptial

I confirm that I have read this submission and believe that I have an appropriate level of expertise to confirm that it is of an acceptable scientific standard.

# Peer review: Reviewer response for version 2

**DOI:** 10.21956/hrbopenres.14835.r32374

**Published:** 2022-06-23

**Authors:** Graham McClelland

**Affiliations:** 1 North East Ambulance Service NHS Foundation Trust, Newcastle upon Tyne, UK; 2 Newcastle University, Newcastle upon Tyne, UK

No further comments

Is the study design appropriate for the research question?

Yes

Is the rationale for, and objectives of, the study clearly described?

Partly

Are sufficient details of the methods provided to allow replication by others?

Yes

Are the datasets clearly presented in a useable and accessible format?

Not applicable

Reviewer Expertise:

Prehospital care and stroke

I confirm that I have read this submission and believe that I have an appropriate level of expertise to confirm that it is of an acceptable scientific standard.

# Peer review: Reviewer response for version 1

**DOI:** 10.21956/hrbopenres.14771.r31725

**Published:** 2022-04-22

**Authors:** Janet E. Bray

**Affiliations:** 1 Department of Epidemiology and Preventive Medicine, Department of Epidemiology and Preventive Medicine, Melbourne, Vic, Australia

**Suggestions:**

1. Title -Suggest using prehospital times and volume rather than “care”.
2. Objective -response times: suggest changing the overall wording from ambulance “response” times to ambulance times -given you are also looking at time to the hospital.
3. Initial and secondary search -seems unusual. Your search strategy is not finalised? Please provide a reference supporting this method of search.
4. The inclusion criteria reads as if a study must include both volume and times, and then all times. Do you mean “or” rather than “and”?
5. Why would you exclude studies only reporting the secondary outcome?
6. Volume: Dispatcher diagnosis (most are not stroke/TIA)  is quite different to paramedic diagnosis (most are stroke/TIA depending on whether a prehospital stroke screen is used). Your analysis suggests you will combine these?  
7. Do you intend to include non-English language studies as stated? How will these be interpreted?
8. How will you handle studies reporting medians or means for times? How will you handle studies reporting adjusted data?
9. The English could be improved throughout (e.g. There has been some research done in the area”; “Consistency of data extraction will be achieved…”).

Is the study design appropriate for the research question?

Yes

Is the rationale for, and objectives of, the study clearly described?

Yes

Are sufficient details of the methods provided to allow replication by others?

Yes

Are the datasets clearly presented in a useable and accessible format?

Not applicable

Reviewer Expertise:

Prehosptial

I confirm that I have read this submission and believe that I have an appropriate level of expertise to confirm that it is of an acceptable scientific standard, however I have significant reservations, as outlined above.

# Response

**Published:** 2022-05-30

**Authors:** Edel Burton

**Affiliations:** University College Cork, Ireland

Thank you for reviewing this protocol. Please find below responses to your comments:

**Title-Suggest using prehospital times and volume rather than “care”.**

 Response:

*Thank you for your comment. We believe this title is appropriate as prehospital care also includes patient transport, which is one of the key outcomes of this review. Furthermore, as time is a crucial component of stroke/TIA care we believe the term “prehospital emergency care” is precise.*

**Objective -response times: suggest changing the overall wording from ambulance “response” times to ambulance times -given you are also looking at time to the hospital.**

*Response:*

*The word response has been “removed” leaving “ambulance times” throughout the protocol, unless we are specifically referring to response time.*

**Initial and secondary search -seems unusual. Your search strategy is not finalised? Please provide a reference supporting this method of search.**

*Response:*

*The approach taken is supported in the attached references:*

***https://libguides.uvic.ca/systematic_reviews_how_to_guide/step1***

***https://libguides.rcsi.ie/systematicreviews/wheretostart***

*The initial search was included in the protocol for the purpose of transparency and replicability. As we are using the Peer Review in Electronic Search Strategies (PRESS) criteria, the search strategy took some time to confirm, at the time of publishing this updated protocol the search strategy is now confirmed, with the help of two librarians.*

*The above references are now added to the initial search section "* ***Initial search ^29,30^: *** ProQuest and PubMed will be used to search for relevant articles."

***The inclusion criteria reads as if a study must include both volume and times, and then all times. Do you mean “or” rather than “and”?***

*Response:*

*Yes, thank you. This has now been amended for clarification.*

*“Studies need to include data on ambulance times or stroke/TIA emergency call volumes in order to be considered for inclusion”. - Inclusion and Exclusion Criteria*

**Why would you exclude studies only reporting the secondary outcome?**

*Response:*

*Thank you, this sentence has now been removed from "the exclusion criteria".*

***Volume: Dispatcher diagnosis (most are not stroke/TIA)  is quite different to paramedic diagnosis (most are stroke/TIA depending on whether a prehospital stroke screen is used). Your analysis suggests you will combine these?***

*Response:*

*The secondary outcome of interest in this reviewer is dispatcher diagnosis. If a study includes paramedic diagnosis than the study will not be excluded. The two types of diagnosis will not be combined but included in a subgroup analysis.*

***Do you intend to include non-English language studies as stated? How will these be interpreted?***

*Response:*

*Yes, we intend to include non-English language studies if appropriate. These will be sent to colleagues in University College Cork or a relevant agency for translation.*

**How will you handle studies reporting medians or means for times? How will you handle studies reporting adjusted data?**

*Response:*

*Thank you, we will contact the author directly and ask for the detail.*

*"If a study is eligible for inclusion in the review but does not include sufficient data for inclusion in the meta-analysis the corresponding study authors will be contacted for access to raw data, in the first instance." - "Presenting and Reporting the Results"*

**The English could be improved throughout (e.g., There has been some research done in the area”; “Consistency of data extraction will be achieved…”).**

*Response:*

*Thank you, amendments have now been made to the use of language in the protocol.*

*“Research in this area has previously focused on the nature of volume emergency medical services calls, prehospital stroke triage  and acute stroke hospital-based care, during the COVID-19 pandemic”. – introduction*

*“EB will extract the data from the included papers. JA* *will check a random sample of  of 20% of these studies for accuracy of data extraction”. – "Data Extraction and Management"*

**

# Peer review: Reviewer response for version 1

**DOI:** 10.21956/hrbopenres.14771.r31723

**Published:** 2022-04-08

**Authors:** Eithne Sexton

**Affiliations:** 1 Data Science Centre, School of Population Health, Royal College of Surgeons in Ireland, Dublin, Ireland

Thank you for this opportunity to review this paper which makes a valuable contribution to the literature on prehospital care for stroke and the impact of Covid on healthcare delivery. In general, the protocol is clear and detailed, making good use of the PRISMA guidelines

 In my view, the protocol could benefit from clarification on the following points:  

1. Selection Criteria – only studies that include all the three definitions of ambulance response time will be included in the study. The feasibility of the review is based on all studies using comparable definitions of response time (albeit with different terminology).  The definitions as given are based on an Irish source. Can you provide some international evidence to support the consistency of these definitions across locations/health systems?
2. Quality appraisal – some more detail on quality appraisal would be helpful. Please justify the choice of the JBI critical appraisal tool for this specific study. What information on quality does it generate (e.g. a score, nominal categories), and how will this be used in the sub-group analysis? The GRADE tool is also mentioned – clarify what this is being used for and how it will add to the information from the JBI tool.
3. Sub-group analysis – How will factors be categorised – e.g. location? income level? It would be useful to pre-specify how these categories are defined, as this is likely to influence the results.  
4. Conclusion - Are these results only relevant to prehospital management of non-communicable diseases during a pandemic? Is there any relevance of the results for prehospital management more broadly? It would be interesting to explore how these results can inform our understanding of healthcare system resilience in response to crisis more generally.   

Is the study design appropriate for the research question?

Yes

Is the rationale for, and objectives of, the study clearly described?

Yes

Are sufficient details of the methods provided to allow replication by others?

Partly

Are the datasets clearly presented in a useable and accessible format?

Not applicable

Reviewer Expertise:

Stroke epidemiology; epidemiological modelling; health services research

I confirm that I have read this submission and believe that I have an appropriate level of expertise to confirm that it is of an acceptable scientific standard, however I have significant reservations, as outlined above.

# Response

**Published:** 2022-05-30

**Authors:** Edel Burton

**Affiliations:** University College Cork, Ireland

Thank you for reviewing this protocol. Please find responses to your comments below:

**Selection Criteria – only studies that include all the three definitions of ambulance response time will be included in the study. The feasibility of the review is based on all studies using comparable definitions of response time (albeit with different terminology).  The definitions as given are based on an Irish source. Can you provide some international evidence to support the consistency of these definitions across locations/health systems?**

*Response:*

*Thank you, the methods section has been amended to reflect the varying terminology and definitions used for ambulance response times internationally. The definitions of “activation time”, “response time”, and “patient care time” were chosen to facilitate comparison, as have been used a previous study. These are key phases in the stroke/TIA patient journey.  Stakeholders from the ambulance service will now also be involved in the review to advise on terminology.*

*“The time periods chosen are due to clinical significance and previous inclusion in ambulance time studies. Due to international variation in terminology and definitions, a standardised definition needed to be used. Members of the ambulance service will advise on this task.”*

*“Members of the Irish National Ambulance Service advised on terminology and clinical significance of time periods, in this review”.*

**Quality appraisal – some more detail on quality appraisal would be helpful. Please justify the choice of the JBI critical appraisal tool for this specific study. What information on quality does it generate (e.g. a score, nominal categories), and how will this be used in the sub-group analysis? The GRADE tool is also mentioned – clarify what this is being used for and how it will add to the information from the JBI tool.**

*Response:*

*Thank you, sentences to clarify this are now added under “ appraisal of the quality of included studies”.*

 “ *The JBI tools will be used as it is anticipated that eligible studies will be cohort studies or quasi-experimental studies. JBI offers a critical appraisal tool for both. The JBI checklist offers a series of questions to which “Yes”, “No”, “Unclear” and “Not applicable” are the provided answers. These checklists will be used to assess risk of bias in individual studies. The GRADE tool is being used to assess the overall quality of cumulative evidence."*

**Sub-group analysis – How will factors be categorised – e.g. location? income level? It would be useful to pre-specify how these categories are defined, as this is likely to influence the results.**

*Response:*

*Thank you, some of the categories can be classified prospectively, whereas others cannot.*

***Location***

 This factor *cannot be categorised prospectively as we are unsure how this will be reported in different papers i.e., some may include cities, states, regions, or countries.*

*“ Classification will be determined once papers are selected.”*

***Income level***

*This will be categorized as low, lower-middle, upper-middle, and high-*income countries *, according to World Bank classifications.*

*“Determined by World Bank Classification ^36^. ^"^*

***Study quality***

*“This will be determined by the appropriate JBI checklist ^33^ critical appraisal tool ^33^criteria.”*

***COVID-19 case numbers/hospitalisations in the country/area at the time of the study.***

*“John Hopkins Coronavirus Resource Centre  Oxford Martin School data will be used.”*

***According to the number of weeks since the World Health Organisation categorized COVID-19 as a pandemic.*** *(This was stated by the Director-General of the WHO at the media briefing on 11 March 2020* *^2^* *)*

***According to stroke/TIA diagnosis***

*This will be classified as suspected stroke/ TIA (call-taker classification), working diagnosis after review by emergency medical services, or hospital confirmed diagnosis.*

**Conclusion - Are these results only relevant to prehospital management of non-communicable diseases during a pandemic? Is there any relevance of the results for prehospital management more broadly? It would be interesting to explore how these results can inform our understanding of healthcare system resilience in response to crisis more generally.**

****

*Response:*

*Thank you for this suggestion, a sentence clarifying same has now been added to the conclusion.*

*“This work may also influence policy guidelines and future  research on prehospital management of non-communicable diseases during a pandemic, and prehospital care more broadly. The results may also inform* *inform our understanding of healthcare system resilience in response to crises on a broader level”.*

# Peer review: Reviewer response for version 1

**DOI:** 10.21956/hrbopenres.14771.r31724

**Published:** 2022-04-04

**Authors:** Graham McClelland

**Affiliations:** 1 North East Ambulance Service NHS Foundation Trust, Newcastle upon Tyne, UK; 2 Newcastle University, Newcastle upon Tyne, UK

This is a protocol for a systematic review and meta-analysis looking at the effect of the COVID-19 pandemic on prehospital care for stroke and TIA. I think this is a worthwhile project but there are some details that need clarification or expanding upon. 

- The title says prehospital emergency care whereas the paper is actually looking at times and call volumes which are not strictly the same so the title could be more precise. 
- The fact that this is looking at adults only could be made clearer earlier on.
- The introduction builds a convincing argument for the impact of COVID-19 on the wider healthcare system but needs to expand on why ambulance response times are so relevant for this population, how brain damage is prevented, and why stroke/TIA are particularly vulnerable to pressures on the health system as these are stated but not explained. 
- Grouping stroke and TIA together needs to be justified as some systems will have different treatment pathways for these conditions.
- The aim talks about estimating the ambulance response time, this seems somewhat vague when these times should be known and clearly reported so can be stated rather than estimated.
- Some definitions of the timeframe being examined would be useful both for how far pre-pandemic and what the authors are classing as the pandemic are needed.
- Are the authors looking at the raw number of stroke calls, how are these being identified (call taker classification, EMS clinician classification), and are these being put into the context of a number of calls to the wider EMS system at the time? 
- Are any underlying trends in times and call volumes pre-pandemic being reported and how will these be accounted for in the analysis?
- I question the use of three time periods as what the authors class as 'patient care time' includes two distinct phases, the at-scene time and the transport to hospital time which are influenced by different factors. 
- Why is hospital diagnosis of stroke being included and how is this relevant to the prehospital times and call volumes which are the primary aims of the study?
- Does a paper need to meet all the inclusion criteria to be included or could papers reporting one or two of the time periods be eligible?
- Does the search have a defined time period, I would assume it would be literature from the last year or two years but this is not stated.
- Will the authors be forward citation chaining to increase the robustness of the search strategy?
- Is there a risk of missing relevant data by restricting the search to peer reviewed journals given the short time frame since the start of the pandemic and the fact that it is potentially not over?
- Why are 3 articles being used to check the consistency of data collection, is there a rationale for this number?
- Will the dispatch systems be reported on as these will affect the response and initial identification of stroke before face to face assessment by EMS clinicians?
- How will the review account for patients assessed by EMS who then did not travel to hospital?

Is the study design appropriate for the research question?

Yes

Is the rationale for, and objectives of, the study clearly described?

Partly

Are sufficient details of the methods provided to allow replication by others?

Yes

Are the datasets clearly presented in a useable and accessible format?

Not applicable

Reviewer Expertise:

Prehospital care and stroke

I confirm that I have read this submission and believe that I have an appropriate level of expertise to confirm that it is of an acceptable scientific standard, however I have significant reservations, as outlined above.

# Response

**Published:** 2022-05-30

**Authors:** Edel Burton

**Affiliations:** University College Cork, Ireland

Thank you for reviewing this protocol. Please see responses to your comments below:

**The title says prehospital emergency care whereas the paper is actually looking at times and call volumes which are not strictly the same so the title could be more precise.**

*Response:*

*Thank you for your comment. We believe this title is appropriate as prehospital emergency care also includes patient transport, which is one of the key outcomes of this review. Furthermore, as a rapid response is a crucial component of stroke/TIA care, due to the time-sensitive nature of intervention and treatment, we believe the term “prehospital emergency care” is precise.*

**The fact that this is looking at adults only could be made clearer earlier on.**

*Response:*

*Thank you, as the population of interest is included throughout the abstract and introduction this clarification has  now been added to the title of the protocol to make this fact clearer earlier on.*

 " *Effects of the COVID-19 pandemic on prehospital emergency* *care for adults with stroke and transient ischaemic attack: A protocol for* a systematic review and meta-analysis"

**The introduction builds a convincing argument for the impact of COVID-19 on the wider healthcare system but needs to expand on why ambulance response times are so relevant for this population, how brain damage is prevented, and why stroke/TIA are particularly vulnerable to pressures on the health system as these are stated but not explained.**

*Response:*

*Thank you, sentences expanding on the above have been added to the introduction.*

*“Ambulance times are relevant to prehospital stroke/TIA care as the role of emergency medical services in this context involves rapid transport to secondary care specialists. As treatment strategies for stroke/TIA are time-dependent it is important to minimise time delays in the prehospital phase of care ^12."^*

*"The acute ischaemic stroke chain of recover y involves recognition (of symptoms), reaction (emergency services are called), response (medical assessment), reveal (brain imaging) and Rx (treatment initiation* ^17^ *."*

*“Due to the time-sensitive nature of stroke/TIA intervention it is imperative that stroke/TIA survivors present to hospital as soon as possible after symptoms develop. ^13^ An increased volume of emergency calls may mean that not as many call takers or ambulances are available. Furthermore, patients may have been hesitant to call an ambulance during COVID-19 due to fear of contracting the disease. ^7"^*

**Grouping stroke and TIA together needs to be justified as some systems will have different treatment pathways for these conditions.**

*Response:*

*Thank you, a justification has been added to the introduction. We wished to include both stroke/TIA as this review is focusing on the symptoms provided to the dispatcher thus no clinical assessment or working diagnosis would have yet been made. Furthermore, as some system s, like AMPDS used code 28 to identify stroke/TIA we wanted to be inclusive of these.*

*“Stroke and TIA can have similar presentations and are being included in this review as it is focusing on the symptoms provided to the dispatcher thus no clinical review or diagnosis would have yet been made. Also, within the timeframe in which prehospital care practitioners care for the patient it may not be possible to differentiate between symptoms of a stroke/TIA. Furthermore, some dispatch systems such as AMPDS have the same code for stroke and TIA.”*

**The aim talks about estimating the ambulance response time, this seems somewhat vague when these times should be known and clearly reported so can be stated rather than estimated.**

*Response:*

*Thank you, we agree that these times should be known and clearly reported, but we cannot be entirely sure whether each study includes the entire population or whether the time data used is directly transcribed from digital systems or estimated by staff on scene. This is the reason that we have used the word ‘estimate’.*

**Some definitions of the timeframe being examined would be useful both for how far pre-   pandemic and what the authors are classing as the pandemic are needed.**

*Response:*

*Thank you, we had mentioned the WHO definition of the pandemic as beginning on 11 ^th^ March 2020 in the  “Presenting and Reporting the Results” section, however have now clarified this also in the introduction. The “pre-pandemic” period will be determined by each article, individually.*

*“COVID-19 was declared a pandemic by the Director-General of the WHO at the media briefing on 11 March 2020 ^2^.”*

**Are the authors looking at the raw number of stroke calls, how are these being identified (call taker classification, EMS clinician classification), and are these being put into the context of a number of calls to the wider EMS system at the time?**

*Response:*

*Thank you, this has been clarified in the inclusion and exclusion criteria. We are looking at call taker classification. If the article provides information on the number of calls to the wider EMS system, then we will put this into context in the review.*

*"Calls identified by the call taker as suspected stroke/TIA will be included* and if the data are available these calls will be put in context of calls made to the wider EMS system."

**Are any underlying trends in times and call volumes pre-pandemic being reported and how will these be accounted for in the analysis?**

*Response:*

*If underlying trends are available in articles these will be reported in the results section. They will be included in a sensitivity analysis if presented.*

**I question the use of three time periods as what the authors class as 'patient care time' includes two distinct phases, the at-scene time and the transport to hospital time which are influenced by different factors.**

*Response:*

*Thank you. If the information is available in the articles, then the two distinct phases of “patient care time” will be included. This is now clarified in the methods and design section*.

*“In this review “patient care time” will include time spent on scene and transport to hospital time.”*

**Why is hospital diagnosis of stroke being included and how is this relevant to the prehospital times and call volumes which are the primary aims of the study?**

*Response:*

*This is included to capture studies which follow the patient through from calling an ambulance to their hospital stay.*

**

**Does a paper need to meet all the inclusion criteria to be included or could papers reporting one or two of the time periods be eligible?**

*Response:*

*Thank you, we have clarified in the inclusion and exclusion criteria that papers need to include one or more of the time periods.*

*“A study needs to include data on at least one of:  activation time, ambulance response time and patient care time to be eligible for inclusion”.*

**Does the search have a defined time period, I would assume it would be literature from the last year or two years but this is not stated?**

****

*Response:*

*Thank you, there is no time period on the search so that it is as inclusive as possible. As COVID-19 was present in some countries earlier than others we wished not to exclude any potentially eligible papers by defining a search time period.*

**Will the authors be forward citation chaining to increase the robustness of the search strategy?**

*Response:*

*Thank you, yes both backward and forward citation chaining searching will be used. Clarification of same has been added to the "reference list search" section.*

*“*Backward and forward citation searching will be conducted on all included studies."

**Is there a risk of missing relevant data by restricting the search to peer reviewed journals given the short time frame since the start of the pandemic and the fact that it is potentially not over?**

*Response:*

*Thank you, peer reviewed literature will provide numeric information related to primary and secondary outcomes for possible inclusion in the meta-analysis. Grey literature, such as abstracts may not include information that is detailed enough to be included in this systematic review and meta-analysis.*

**Why are 3 articles being used to check the consistency of data collection, is there a rationale for this number?**

****

*Response:*

*This has now been changed to 20% in the "Data Extraction and Management" section, which is commonly seen in systematic reviews.*

*"JA will check a random sample of 20% of these studies for accuracy of data extraction.”*

**Will the dispatch systems be reported on as these will affect the response and initial identification of stroke before face-to-face assessment by EMS clinicians?**

*Response:*

*Thank you for this suggestion, we will now report the dispatch systems. This has been added to table **3.***

**How will the review account for patients assessed by EMS who then did not travel to hospital?**

*Response:*

*Thank you, as the inclusion criteria have now been changed to capture at least one of the time periods of interest, patients who did not travel to hospital will be captured.*
